# Microcephaly in Infants, Pernambuco State, Brazil, 2015

**DOI:** 10.3201/eid2206.160062

**Published:** 2016-06

**Authors:** Microcephaly Epidemic Research Group

**Keywords:** Microcephaly, infants, viruses, disease outbreaks, Brazil, Zika virus, vector-borne infections

## Abstract

We studied the clinical characteristics for 104 infants born with microcephaly in the delivery hospitals of Pernambuco State, Brazil, during 2015. Testing is ongoing to exclude known infectious causes. However, microcephaly peaked in October and demonstrated central nervous system abnormalities with brain dysgenesis and intracranial calcifications consistent with an intrauterine infection.

^1^Members of the Microcephaly Epidemic Group who contributed data are listed at the end of this article.

In April 2015, Zika virus was identified in Brazil (*1*,*2*). In August, an increased incidence of microcephaly was detected in Pernambuco State. In November, the Brazilian Ministry of Health declared a relationship between the microcephaly epidemic and Zika virus infection during pregnancy (*3*) on the basis of accumulating evidence (Technical Appendix). Since then, several reports of Zika virus–associated microcephaly have been published (*4*–*6*). As of December 12, 2015, a total of 2,401 suspected cases of microcephaly had been reported (including 29 stillbirths) in 549 municipalities in 20 states in Brazil; Pernambuco reported the most (874 cases) (*7*). In comparison, an annual mean of 156 microcephaly cases were reported in Brazil (through the routine birth notification system) during 2010–2014, including 9 in Pernambuco (*7*). We describe preliminary findings of 104 microcephaly cases in Pernambuco from the 2 hospitals to which infants with suspected cases were referred.

## The Study

In August 2015, we began systematically collecting data prospectively and retrospectively (by reviewing hospital records dating to April 2015) on infants suspected to have microcephaly and referred to us. We defined microcephaly as head circumference below the third percentile for gestational age and sex using the Fenton growth chart (*8*). Our routine protocol for reviewing suspected microcephaly cases involved collecting data on maternal age and infant sex, gestational age, and birthweight. Laboratory testing was performed for 6 pathogens: serologic testing for dengue virus; and a nontreponemal test for toxoplasmosis, rubella, cytomegalovirus, herpes simplex virus, and syphilis (collectively referred to in Brazil as ToRCHeS agents). Zika virus testing was not available at the time of the study. Brain imaging was performed when available by using computed tomography (CT) scans, magnetic resonance imaging, or transfontanellar ultrasonography. Hearing was assessed by using otoacoustic emission testing, and retinae were examined by using handheld fundoscopy. We asked mothers about rash during pregnancy, but no data were collected on the timing of the rash, other clinical symptoms, or environmental exposures. We excluded patients with suspected microcephaly who had a head circumference greater than the third percentile (40 infants), were missing chart data for head circumference (6 infants) or gestational age (4 infants), or tested positive for a congenital infection known to cause microcephaly (1 infant positive for cytomegalovirus identified by PCR). We plotted included cases by week of birth and compared variables of infants with microcephaly (head circumference 30–32 cm) and those with severe microcephaly (circumference <30 cm). We conducted this investigation as part of our routine clinical practice; it did not require human subject approvals.

**Figure 1:**
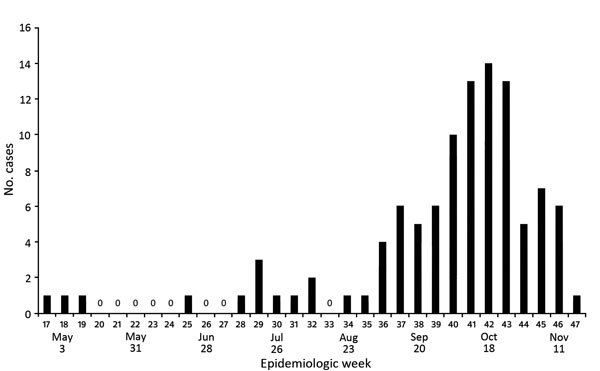
Microcephaly cases (based on Fenton growth chart criteria) at 2 referral hospitals, by week of birth, Pernambuco State, Brazil, 2015 (N = 104).

**Figure 2:**
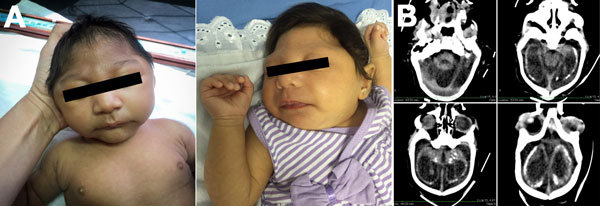
Microcephaly, Pernambuco State, Brazil, 2015. A) Two newborns in whom microcephaly was diagnosed during the epidemic. B) Brain computed tomography scan of a 43-day-old infant showing cerebellar hypoplasia, parenchymal calcifications, ventriculomegaly, and malformation of cortical development compatible with lissencephaly.

Our final study comprised 104 infants with microcephaly. Cases increased from epidemiologic week 37 (mid-September) and peaked during weeks 40–43 (late October) (Figure 1). Seventy infants had severe microcephaly (Table; Figure 2, panel A) and a mean head circumference of 29 cm. Only 10% of case-infants were born prematurely, which was lower than the national estimates of the prevalence of premature birth (*9*). Mothers were a mean of 25 (range 15–43) years of age. Of the 100 mothers interviewed, 59 recalled having a rash during pregnancy. Testing for dengue and ToRCHeS was incomplete for more than half of the case-infants; the number tested for each of the 6 pathogens varied because of limited laboratory resources at the beginning of the epidemic (Table). Three infants tested positive for syphilis, 2 for dengue, and 1 for herpes simplex virus, but they also shared common features with the epidemic of congenital microcephaly (*10*).

**Table:** Characteristics of 104 newborns with microcephaly seen at 2 referral hospitals, Pernambuco State, Brazil, August–December 2015*

Finding	Severe microcephaly, no. positive/no. tested (%)	Microcephaly, no. positive/no. tested (%)	p value†
Mother			
Gestational age of newborn, N = 104			
At term, 37 wks to 41 wks 6 d	59/70 (84)	34/34 (100)	0.019
Preterm, <37 wks	10/70 (14)	0/34 (0)	
Postterm, >42 wks	1/70 (1)	0/34 (0)	
Self-reported rash during pregnancy, n = 100	41/68 (60)	18/32 (56)	0.702
Newborn‡			
Female sex, N = 104	38/70 (54)	16/34 (47)	0.489
Brain CT scan or MRI, n = 58			
Calcifications	43/45 (96)	11/13 (85)	0.208
Malformation of cortical development, including lissencephaly	32/45 (71)	8/13 (61)	0.517
Ventriculomegalia	32/45 (71)	6/13 (46)	0.102
Abnormal findings in transfontanellar ultrasound, n = 32	23/23 (100)	6/7 (86)	0.092
Abnormal findings in fundoscopy,§ n = 33	8/29 (28)	0/4 (0)	0.550
Abnormal findings in OAE,§ n = 23	2/16 (12)	0/7 (0)	1.000
Newborn serology			
Nontreponemal syphilis test, n = 62	2/42 (5)	1/20 (5)	1.000
Dengue virus IgM, n = 34	2/25 (8)	0/9 (0)	1.000
Cytomegalovirus IgM, n = 33	0/24 (0)	0/9 (0)	NA
Cytomegalovirus IgG, n = 34	19/23 (83)	8/11 (73)	0.245
Toxoplasmosis IgM, n = 44	0/37 (0)	0/7 (0)	NA
Toxoplasmosis IgG, n = 47	24/39 (61)	5/8 (62)	1.000
Rubella IgM, n = 54	0/42 (0)	0/12 (0)	NA
Rubella IgG, n = 88	55/56 (98)	28/32 (87)	0.396
Herpes IgM, n = 19	1/15 (7)	0/4 (0)	1.000
Herpes IgG, n = 18	13/14 (93)	4/4 (100)	1.000

*Severe microcephaly, head circumference <30 cm; microcephaly, head circumference 30–32 cm. CT, computed tomography; MRI, magnetic resonance imaging; NA, not applicable; OAE, otoacoustic emission test;. †χ^2^ comparing frequencies between the 2 groups. ‡Of the 98 infants with documented birthweight, average weight was 2,716 g (range 1,630–3,890 g) for term and postterm newborns and 1,918 g (range 1,135–2,580 g) for preterm. §Of the 33 children examined by fundoscopy, 24% had abnormal findings, including rarefaction of the retinal pigment epithelium, atrophic macular lesions, macular scarring and macular reflection juxtafoveal changes. Of the 23 children tested with OAE, 9% had an abnormal result. The frequency of abnormal results in preterm vs. term and postterm newborns for OAE and fundoscopy was similar (<30%).

As of January, 13, 2016, a total of 58 of the 104 infants had been investigated (54 by CT scan, 3 by magnetic resonance imaging, 1 by both). All 58 infants showed radiologic abnormalities, including calcifications (93%), mainly in the cortical/subcortical junction but also in the periventricular region, basal ganglia, thalamus, midbrain, and cerebellum. Sixty-nine percent showed evidence of malformations of cortical development, including lissencephaly (relative smoothness of the brain surface) associated with pachygyria (poorly formed, broad cortical folds), agyria (no cortical folds), or both (Figure 2, panel B). Sixty-six percent had ventriculomegaly, an enlargement of the ventricles that can occur for several reasons, such as loss of brain volume or impaired outflow or absorption of cerebrospinal fluid from the ventricles. Of the 15 children with pending CT scans who had undergone ultrasonography, 14 had abnormalities, such as calcifications and brain atrophy. The child (at term) with a normal ultrasound had a head circumference of 30 cm, and the mother reported a rash during pregnancy.

## Conclusions

Microcephaly among the 104 newborns peaked in October 2015 and demonstrated severe central nervous system abnormalities with brain dysgenesis and intracranial calcifications consistent with an intrauterine infection. Although we initially lacked Zika virus testing and completed only partial testing for ToRCHeS infections, the timing on the microcephaly and the history of rash in more than half of the pregnant mothers suggest an outbreak of congenital microcephaly caused by a congenital infection. Since our study was completed, less clinically detailed and more surveillance-oriented data have been published from the national reporting system (*11*). These data include the case-infants reported here and the reported occurrence of the 2015–2016 microcephaly cases, especially in Pernambuco. They highlight the temporal relationship between the preceding Zika virus transmission and the abrupt increase in prevalence of microcephaly at birth.

Our study focused on the clinical findings of 1 cohort, demonstrating a high proportion of calcifications and malformations of cortical development in infants with microcephaly and with severe microcephaly. Such malformations have commonly been observed in cases of intrauterine infections caused by cytomegalovirus and toxoplasmosis (*12*); more severe anomalies (e.g., lissencephaly) occur in infants whose mothers were infected before weeks 16–18 of pregnancy (*13*). The finding of intracranial calcifications predominantly in the cortical/subcortical parenchyma suggests a scan pattern distinct from that of other congenital infections, although further studies including comprehensive diagnostic testing for Zika virus and other known infectious causes of congenital central nervous system defects are needed to confirm this pattern.

Our study had several limitations. First, as previously noted, this study was preliminary, and Zika virus testing was not yet available. Second, personnel and laboratory resources were insufficient for ToRCHeS testing of more than half of the case-infants reported here; however, we collected blood and cerebrospinal fluid samples for future testing. Third, limited resources restricted the number of brain scans to slightly more than half of these infants. Fourth, the data on rash during pregnancy was collected postpartum and could be subject to recall bias. Nevertheless, a case–control study has been under way in Pernambuco since December 2015, supported by the Brazilian Ministry of Health and the Pan American Health Organization, to establish an association between microcephaly and Zika virus (primary hypothesis) and explore other infectious or noninfectious causes.

Although the ToRCHeS testing was incomplete, our findings suggest an outbreak of severe microcephaly in Pernambuco that peaked in October 2015. Our data, in conjunction with recent surveillance summaries from Brazil (*11*), are consistent with the timing of the Zika virus epidemic. Our findings illustrate the most severe end of the spectrum of defects affecting newborns. Other manifestations and complications in infants born to mothers infected with Zika virus during pregnancy will be described through close follow-up of these children. The government of Brazil plans to expand the support system for affected children. Further studies will define the proportion of congenital defects according to the gestational age of infection and describe the clinical outcomes.

Technical Appendix Accumulating evidence of a relationship between microcephaly and Zika virus infection during pregnancy.
