# Supplementary material for: Microcephaly in Infants, Pernambuco State, Brazil, 2015
Source: Emerg Infect Dis. 2016 Jun;22(6):1090–3. doi: 10.3201/eid2206.160062 (PMC4880105; doi:10.3201/eid2206.160062)
Supplement: Technical Appendix — Accumulating evidence of a relationship between microcephaly and Zika virus infection during pregnancy. [file 16-0062-Techapp-s1.pdf]

Article DOI: http://dx.doi.org/10.32032/eid2206.160062

Microcephaly in Infants, Pernambuco, Brazil, 2015

Technical Appendix

Accumulating Evidence of a Relationship between Microcephaly and Zika Virus Infection during Pregnancy

Evidence is accumulating of the link between Zika virus infection and microcephaly. A high proportion of mother of newborns with microcephaly reported fever and rash during pregnancy (*1*). The timing of the microcephaly epidemic was roughly 6 months after detection of an epidemic of fever and rash attributed to Zika virus (*1*). The disperse geographic distribution of microcephaly cases was not compatible with toxic exposure (*1*). Brain images of affected newborns showed calcifications and other anomalies suggestive of an infectious origin in morphologic studies (*2*). There was lack of laboratory evidence for established causes of congenital infections (grouped under ToRCHeS: toxoplasmosis, parvovirus, rubella, cytomegalovirus, herpes, syphilis) in most of the cases, in the preliminary investigation (*3*). Zika virus was identified in amniotic fluid of 2 women in which microcephaly was diagnosed in the fetus and in the brain tissues of a newborn with microcephaly who died soon after birth (*1*,*4*,*5*).

References

1. Brazilian Ministry of Health. Protocol for monitoring and response to microcephaly occurrence relating to ZikaV infection [in Portuguese] [cited 2015 Dec 9]. http://portalsaude.saude.gov.br/images/pdf/2015/dezembro/09/Microcefalia-Protocolo-de-vigil–ncia-e-resposta-vers-o-1--09dez2015-8h.pdf

2. Mlakar J, Korva M, Tul N, Popović M, Poljšak-Prijatelj M, Mraz J, et al. Zika virus associated with microcephaly. N Engl J Med. 2016;374:951–8. PubMed http://dx.doi.org/10.1056/NEJMoa1600651

3. de Oliveira WK, Coelho GE, de França GVA. Boletim epidemiológico—situação epidemiológica de ocorrência de microcefalias no Brasil, 2015. Bol Epidemiológico da SVS/MS. 2015;46(34):1–3

[cited 2015 Nov 19]. http://portalsaude.saude.gov.br/images/pdf/2015/novembro/19/Microcefalia-bol-final.pdf

4. Pan American Health Organization. Neurological syndrome, congenital malformations, and Zika virus infection. Implications for public health in the Americas. Epidemiological Alert [cited 2015 Dec 1]. http://www.paho.org/hq/index.php?option=com_docman&task=doc_view&Itemid=270&gid=32405&lang=en

5. Calvet G, Aguiar RS, Melo AS, Sampaio SA, de Filippis I, Fabri A, et al. Detection and sequencing of Zika virus from amniotic fluid of fetuses with microcephaly in Brazil: a case study. Lancet Infect Dis. 2016;pii: S1473-3099(16)00095-5.

Microcefaly Epidemic Research Group–MERG affiliations:

The Research Center Aggeu Magalhães/Oswaldo Cruz Foundation (Fiocruz), Recife, Brazil (M.F.P.M. Albuquerque, C. Braga, S.P. Brandão-Filho, C.A.A. Brito, M.T. Cordeiro, R. Dhalia, E.T.A. Marques, C.M.T. Martelli, W.V. Souza); Federal University of Pernambuco, Recife, Brazil (T.V.B. Araújo, R.A.A. Ximenes); University of Pernambuco, Recife, Brazil (M.D.C.G. Carvalho, D.B. Miranda-Filho, V.V.D.L. Mota, R.C.F. Ramos, M.A.W. Rocha, P.F.S. Silva, R.A.A. Ximenes); The Professor Fernando Figueira Integral Medicine Institute, Recife, Brazil (A.S.C. Cunha, D.D.C.S. Cruz, A.N. Hazin, M.M.C. Moraes, A. Van Der Linden); Pernambuco State Health Department (SES-PE), Recife, Brazil (A. Barkokebas, L.C.A. Bezerra, R.G. Cabral, A.R. Carneiro, I. Costa-Jr, L. Katz, C. Mota); Federal University of Rio Grande do Sul, Porto Alegre, Brazil (L. Schüler-Faccini); Federal University of Fronteira Sul, Chapeco, Brazil (J.D.L. Batista); The Institute for Health Technology Assessment, Porto Alegre, Brazil (M.F.P.M. Albuquerque, J.D.L. Batista, M.C. Braga, C.M.T. Martelli, D.B. Miranda-Filho, W.V. Souza, R.A.A. Ximenes); London School of Hygiene & Tropical Medicine, London, UK (L.C. Rodrigues); Pan American Health Organization Brazil, Brasilia, Brazil (J.J. Cortez-Escalante, C.F.C.A. Melo, E. Vazquez); Pan American Health Organization/World Health Organization, Washington D.C., USA (S. Aldighieri, M. Almiron, J. Mendez-Rico, P. Ramon-Pardo); Brazilian Ministry of Health, Brasilia, Brazil (G.E. Coelho, E. Duarte, C.M.P. Henriques, W.K. Oliveira); University of Pittsburgh, Pittsburgh, USA (E.T.A. Marques).
